# Living With Multiple Sclerosis: The Rainbow of Inspiring Experiences and Resilience in the Face of the Disease

**DOI:** 10.1111/hex.70044

**Published:** 2024-10-02

**Authors:** Leily Zare, Nahid Dehghan Nayeri, Fatemeh Bahramnezhad, Arezoo Rasti

**Affiliations:** ^1^ Department of Medical Surgical School of Nursing and Midwifery, Tehran University of Medical Sciences Tehran Iran; ^2^ Nursing and Midwifery Care Research Center, School of Nursing and Midwifery Tehran University of Medical Sciences Tehran Iran; ^3^ Department of/Basic Sciences/Medical Surgical Nursing School of Nursing and Midwifery, Tehran University of Medical Sciences Tehran Iran

**Keywords:** experience, hope, multiple sclerosis, qualitative research, resilience

## Abstract

**Introduction:**

Multiple sclerosis (MS), a leading cause of disability in young adults worldwide, including in Iran, affects their whole life so common care is no longer effective. In this regard, context‐based approaches should be considered for a holistic care delivery that accords with the patients' inputs. We aimed to explore patients' understanding of MS and their personal experiences of living with this disease.

**Methods:**

A qualitative descriptive study was conducted. The data were collected through in‐depth, semi‐structured interviews with 17 patients. These patients were selected using a purposive sampling method, and the data were analyzed using a conventional content analysis approach.

**Findings:**

Three main categories and nine subcategories were identified: Thunder and Lightning strike in the form of Displeasure, Social wrong beliefs, Experiences of Constraints, Interference with Life Stages and Dark Spots on the Horizon of the Future; Subtle Beam consisting of Extrinsic Light Radiation, Reflection of Individual Effort and Formation of a Rainbow by Resilience and Hope for a Bright Future.

**Conclusion:**

By offering multidimensional support, patients reported a shift from fear to a vibrant life. Although research often focuses on the negative aspects of MS, this study recognizes both positive and negative aspects. These findings can contribute to future interventional research.

**Patient or Public Contribution:**

During the explanation of research goals and consent acquisition, participants were reminded that sharing their experiences could provide valuable insights benefiting others coping with or at risk of the same disease. Additionally, during data analysis, codes extracted were reviewed and improved with active participant involvement.

## 1 Introduction

Multiple sclerosis (MS) is a common chronic inflammatory disease of the central nervous system. It usually begins in a person's third or fourth decade of life and is the second leading cause of neurological disability in young adults after injury. According to the 2020 Atlas of MS Disease report, approximately 2.8 million people globally are affected by MS, with 75,000 in Iran [1]. The number of cases is rising both globally and in Iran, with higher prevalence in cities like Tehran and Ardabil. MS has significant social and economic impacts, requiring lifelong support and care [2, 3, 4].

Most studies have quantitatively focused on identifying problems faced by patients and their severity. However, quantitative approaches are limited in understanding the psychological, social and cultural aspects of patients' issues. In contrast, qualitative methods offer a more comprehensive view of patients' experiences. Although some qualitative studies exist, they typically concentrate on specific life stages—such as diagnosis, relapse or recovery—along with particular outcomes and types of MS.

The present study aims to uncover both hidden and explicit patterns to address the questions, ‘How do people with MS experience their illness?’ and ‘How do patients understand their disease?’ This can provide healthcare providers with essential insights into the challenges of living with MS and assist in developing innovative and effective care interventions.

## 2 Methods

### 2.1 Design

This qualitative study aimed to explore patients’ understanding of MS and their personal experiences with the disease. A conventional content analysis approach was employed, avoiding subjective assumptions or preconceived theories. This study was addressed in line with the Consolidated Criteria for Reporting Qualitative Research (COREQ) [5] guidelines (Supporting Information S1: File 1).

### 2.2 Participants and Setting

Participants with diverse demographics were selected through purposive sampling from MS clinics in Tehran and Ardabil (Iran) (Table 1). Inclusion criteria for this study were as follows: having any MS type (Relapsing–Remitting, Primary Progressive, Secondary Progressive or Progressive–Relapsing), being between 18 and 60 years old, receiving treatment at an MS clinic, being able to read and speak Persian and being willing to share experiences.

**Table 1:** Demographic characteristics of participants in this study.

Participants	Sex	Age (year)	Marital status	Education level	Time of diagnosis	Type of MS
1	Female	37	Married	PhD	15 years	Secondary progressive
2	Female	23	Single	BSc	1 year	Primary progressive
3	Female	23	Married	Diploma	2 years	Remitting–Relapsing
4	Female	33	Married	BSc	10 months	Remitting–Relapsing
5	Female	26	Married	High school	10 years	Remitting–Relapsing
6	Female	30	Married	BSc	11 years	Remitting–relapsing
7	Male	43	Married	Middle school	4 years	Remitting–Relapsing
8	Male	32	Married	BSc	3 years	Primary progressive
9	Male	35	Married	Diploma	12 years	Remitting–Relapsing
10	Male	40	Divorced	High school	22 years	Secondary progressive
11	Female	50	Widowed	Elementary	15 years	Remitting–Relapsing
12	Female	48	Married	Elementary	9 years	Remitting–Relapsing
13	Female	48	Married	BSc	16 years	Remitting–Relapsing
14	Female	35	Married	Middle school	3 years	Remitting–Relapsing
15	Female	46	Married	Middle school	9 years	Remitting–Relapsing
16	Female	40	Married	Elementary	6 years	Remitting–Relapsing
17	Female	48	Married	Illiterate	4 years	Remitting–Relapsing

The first participant was chosen as a key informant due to their extensive experience with the disease, longer illness duration and strong ability to articulate experiences and emotions. Participant selection continued until data saturation was reached, indicating that no further valuable insights could be gained. A total of 17 participants were selected to participate, all of whom agreed to be interviewed.

### 2.3 Data Collection and Interviews

Data collection began in late April and continued until late October 2023, involving in‐depth interviews that began with open‐ended questions in an unstructured manner, followed by a semi‐structured approach by the primary author under the research team's supervision. The interviewer was a female, PhD candidate in Nursing with years of experience as an intensive care nurse. She was trained in qualitative research and conducting interviews. The study supervisors were faculty members in Nursing with prior experience in qualitative research. Drawing from the researchers' expertise in qualitative studies on MS patients [6, 7], the interview questions were designed accordingly. The interviews started with general questions about personal characteristics and transitioned to specific topics related to the research objective. All interviews were audio recorded with the consent of the participants. Participants shared their experiences with MS, discussed challenges faced since diagnosis and explained how they addressed them. Probing questions were used throughout, and at the end of each interview, participants could share any additional pertinent information. In total, 17 Interviews were conducted face to face in quiet environments, such as hospital conference rooms or empty facility rooms with no one present besides the interviewer and the participant. On average, the interviews lasted 50–90 min, with an average duration of 45 min.

### 2.4 Data Analysis

The conventional content analysis method was utilized by following the five steps proposed by Graneheim, Lindgren, and Lundman [8] for data analysis. These steps are widely accepted in research due to their user‐friendly nature: (1) Transcribing the entire interview immediately after each interview, (2) Reading the entire text several times to gain an overall understanding of its content, (3) Determining semantic units and basic codes, (4) Classifying primary codes into more comprehensive categories and (5) Determining the main theme of categories. The data collection and data analysis phases continued until data saturation was reached. MAXQDA10 software was used to handle the large volume of data.

### 2.5 Patient or Public Involvement

While explaining the research objectives and obtaining participant consent, it was emphasized that sharing their experiences could provide valuable insights for others facing the same disease. Additionally, during data analysis, the extracted codes were reviewed and refined with the active participation of some participants.

### 2.6 Trustworthiness

To ensure the quality of our study, we applied the criteria proposed by Lincoln and Guba (1985) [9], including credibility, dependability, confirmability, transferability and authenticity. Study credibility was established through prolonged engagement with participants and data, allocating sufficient time for data collection, building trust‐based relationships with participants, reviewing the findings by two participants (R.M. and P.M.) and incorporating their feedback. Dependability was ensured through research auditing and data control by three independent authors, and the extracted codes were reviewed by the research team. Then, the categories were compared with each other. Any disagreement was resolved by discussion. Moreover, a number of interviews were randomly selected and given to some researchers (M.Sh. and F.K.) who were familiar with qualitative research but were not part of the study to review the data and share their comments and feedback. Confirmability and authenticity were guaranteed by presenting quotes as extracted from each interview. Transferability was enhanced by detailing the entire research process, all participants' characteristics and research context and by selecting participants with maximum diversity from two distinct cities, which could also help other researchers to follow the research procedure.

### 2.7 Ethical Consideration

This study is part of an approved mixed‐method research project for a doctoral dissertation that has been ethically approved by the Ethics Committee of Tehran University of Medical Sciences with the number IR.TUMS.FNM.REC.1401.185. All participants provided written informed consent before taking part in this study. They were briefed by the interviewer on the research objectives, their option to withdraw at any point and the schedule for the interview sessions. Consent was also obtained for recording the interviews, which were later deleted after transcription. The interview transcripts were securely stored with measures in place to uphold confidentiality.

## 3 Findings

After the data analysis, three main categories and nine subcategories emerged (Table 2).

**Table 2:** Main categories and subcategories emerged from interviews.

Main categories	Subcategories	Meaning unit
Thunder and Lightning strike	Displeasure	If my doctor had prescribed me the right medicine, I would still be able to walk.
	Social wrong beliefs	The biggest problem with MS for me was having the title ‘Rare Patient’ next to my name.
	Experiences of constraints	I was in a bad mood. I was constantly crying, wailing and lamenting why I had become like this.
	Interference with life stages	I was diagnosed exactly when I was engaged. It was shocking for us.
	Dark spots on the horizon of the future	I am afraid that I will become wheelchair‐bound in the future.
Subtle Beam	Extrinsic light radiation	If you have financial support, you can buy your medicines, follow up your treatment and control your disease.
	Reflection of individual effort	Everybody has the ability to improve his/her situation. Therefore, s/he should start overcoming difficulties with will.
Formation of a Rainbow	Resilience	Now, after months of grounding, I relish every step I take like a baby just learning to walk.
	Hope for a bright future	As a mother, I have a lot of hope for the future. I'm sure I'll get better.

### 3.1 Thunder and Lightning Strike

Thunder and lightning strike is a symbolic representation of the problems and challenges experienced by patients. They voiced several obstacles related to living with MS, leading to secondary challenges in their day‐to‐day activities. These include subcategories of displeasure, Social wrong beliefs, experiences of constraints, interference with life stages and dark spots on the horizon of the future.

#### 3.1.1 Displeasure

Some participants expressed dissatisfaction with the progression of the disease, the treatment process, as well as the actions of their own, families and caregivers.

They were upset with the sudden onset of their disease, which came as a shock when they received an incurable diagnosis. They also expressed frustration that the disease could have started with mild symptoms. Some patients were unhappy with the recurring attacks they experienced over a year.

Certain participants were sceptical about the treatment process, particularly the delay in receiving a diagnosis despite seeking immediate medical attention when the first symptoms appeared. They underwent numerous diagnostic tests and even sought treatment in more advanced medical facilities. These individuals were disappointed to have to rely on modern medicine for treating the disease and suspected the existence of a pharmaceutical mafia.

Some participants expressed dissatisfaction with own actions before and after being diagnosed with MS. They mentioned delay in seeking medical help when symptoms first appeared, not consistently taking their medication and not joining the MS Society as challenges they faced when dealing with the disease or as factors that worsened their MS‐related issues.

During their battle with this disease, some participants mentioned dissatisfaction with the performance of their caregivers. This included their spouses’ indifference and lack of cooperation in household and childcare duties, family members’ misinformation about the contagiousness of the disease, marital distancing, misdiagnosis, delayed diagnosis and inappropriate drug prescriptions.

I know modern medicine has no intention of treating this disease at all. There is a pharmaceutical mafia in the world. Because of that, I am not very hopeful that a new drug will come to cure my disease.

(P 7)

#### 3.1.2 Social Wrong Beliefs

Participants in this study revealed that there are societal taboos surrounding illness, including the word ‘MS’. The negative perceptions attached to this disease along with the fear of being judged for inevitable paralysis lead to the feelings of unease about missed life opportunities and more importantly marriage opportunities.

My illness was a burden for me. MS, the name alone is daunting. People would react with fear, saying oh God, MS leads to paralysis! I told everyone around it was an infection in my head that caused weakness in my legs. Otherwise, their sights only worsened my condition.

(P 9)

#### 3.1.3 Experiences of Constraints

Participants in this study expressed various challenges they faced, including physical constraints such as concentration disorders, exacerbation of symptoms under stress, balance issues, visual impairments, bowel and bladder control problems, unusual fatigue, premature menopause and sexual difficulties.

Psychological limitations such as feelings of despair, mood disorders, depression and apathy were also reported.

Socio‐economic limitations included social isolation, dropping out of school, spending savings on treatment and lack of access to services and medications due to living in remote rural areas.

I used to have my own business and could easily manage my bank accounts, but now my hands shake so much that I can't even sign my checks.

(P 11)

I have completely isolated myself from social life. I was a university lecturer, but now I'm mostly homebound and almost a recluse.

(P 7)

#### 3.1.4 Interference With Life Stages

According to the participants, one of the challenges experienced during the onset of MS and living with this disease was its interference with the developmental stages of life. This includes dealing with the disease when youth strive to establish their career, education or marital life in their vibrant period of lives.

This disease hit me right at the peak of my activity. Just when I felt I was doing my bests, wanting to find a job, expand my social relationships. I had absolutely no problems, suddenly MS hit me like a crisis. I was doing really well in my studies, planning to become a family counselor.

(P 5)

#### 3.1.5 Dark Spots on the Horizon of the Future

Participants expressed concerns about the future, including fears of exacerbating physical problems, dependency, job loss and spousal betrayal. Many also mentioned suicidal thoughts in the face of worsening symptoms or the challenges of the disease.

If I don't get rehabilitated, I can't continue my life under these conditions. If I lose hope, I'll find a solution and commit suicide.

(P 14)

### 3.2 Subtle Beam

Patients listed factors in their lives that help them tolerate the hardships of the disease, enabling them to reduce its limitations. Accordingly, the subtle beam symbolizes all resources that support patients with MS, including subcategories of extrinsic light radiation and reflection of individual effort.

#### 3.2.1 Extrinsic Light Radiation

Participants consider some essential expectations for managing their illness, consisting of support from family, colleagues and MS society as well as financial, informational and counselling assistance. Participants also have expectations that can improve their ability to cope with MS. These include conducting counselling sessions with family members by the treatment team, normalizing MS in society to reduce fear and stigma and increasing understanding among peers.

MS should be normalized in the society. When I was first diagnosed, it was seen as a terrifying disease. It may still be scary for many. Providing information is crucial. Many crises arise from a lack of information. Individuals must be accompanied; they need support and interaction. Emotional and financial assistance is essential. It's important to accommodate their treatment commitment.

(P 5)

#### 3.2.2 Reflection of Individual Effort

In the participants' statements, individual characteristics and actions such as maintaining a positive attitude, being social, believing in the wisdom of God in life matters and coping with life's challenges were highlighted as important factors. Additionally, their efforts in adopting self‐care strategies, such as seeking information from preferred sources and adhering to medication alongside complementary therapies, were mentioned as internal facilitators.

Emotional state is crucial. I do not get caught up in negative things and always try to uplift my mood. I've always been in a good mood. I used to listen to music and dance. Even after being diagnosed with MS, I'm still hopeful. Some people feel hopeless and constantly worry about becoming disabled. I am not so. I always believe I will get better.

(P 9)

### 3.3 Formation of a Rainbow

Patients have shown significant improvement in fighting the illness, providing great motivation to live a normal life despite the challenges of MS. This category symbolizes the consequences experienced by patients living with MS, including subcategories of resilience and hope for a bright future.

#### 3.3.1 Resilience

Resilience involves the recovery of capabilities due to psychosocial changes, such as finding peace, appreciating good health, and adjusting lifestyle habits. Patients learn to perform tasks independently and break free from dependency. The growth and development of individuals with MS include enhancing existential capacities, such as increasing hope for life by focusing on the positive aspects of living with MS, adaptation by trying to forget about the disease and coping through self‐awareness after overcoming challenges. Furthermore, patients find meaning in life by seeking supernatural guidance, such as turning to divination for insight into the future course of the disease, and setting personal boundaries, like choosing not to disclose their illness to others and keeping relatives unaware of their condition.

Finally, a person becomes friends with this disease. They understand each other and get along. You have no choice but to come along with it. The more you bother yourself, the more disease bothers you.

(P 8)

#### 3.3.2 Hope for a Bright Future

Some participants expressed optimism about the future. Contrary to the common belief about the permanence of disabilities, they believe that improvements will eventually be made.

Contrary to people's beliefs about MS, I believe I will get better and I am very hopeful. Right now, my hope for life has increased even more than ever.

(P 5)

## 4 Discussion

We conducted a mixed‐method study. This qualitative content analysis was the first phase of the study aiming to shed light on MS patients' understanding and personal experiences of living with this disease. Through interviews, three main categories were found: thunder and lightning strike, subtle beam and formation of a rainbow, which can be likened to the phenomenon of rainbow formation.

The thunder and lightning symbolize the hardships that patients encounter while dealing with MS. These challenges have an intense impact on the patients' lives, overshadowing their happiness and causing harm. Patients feel frustrated not only with the disease and its treatment but also with their own selves and others. They also struggle against the oppressive beliefs of the illness, face limitations in various aspects of their lives and go through disruptions throughout different stages of life. Ultimately, they perceive looming hurdles in their future, representing their ongoing battles.

Similar studies have highlighted a range of difficulties faced by MS patients, encompassing physical and psychological challenges, uncertainties about what lies ahead, functional restrictions, unemployment, financial strains, familial pressures, the challenges of managing the illness and its treatment and societal misconceptions [10, 11]. However, some other studies have specifically focused on psychological issues [2, 12]. These variations may stem from variations in participants' demographics, social contexts, type and phase of the disease, duration of the condition and research goals. In a study contrary to the current one, MS‐related issues were seen as catalysts for patients to adapt and amend their lifestyles [13]. However, in this study, facing the challenges of the disease led to encountering even more obstacles. This discrepancy may be attributed to the considerable 8‐year gap between data collection and analysis in the mentioned study. Moreover, in that qualitative study, data were collected from patients' online responses to 163 open‐ended questions, which differ from ours. Supporting this study, a review study suggests that the onset of MS symptoms contributes to negative experiences such as worries and disturbances in patients' personal lives [14], hinting at shared stressors for individuals grappling with MS.

The Subtle Beam, the second category, represents supportive resources that consist of external and internal support. Following storms of difficulties, a faint glimmer of light gradually dispels the darkness, ushering in brightness and a sense of renewal. This light symbolizes the vitality derived from these supportive resources. When examining the experiences of individuals with MS, the support they receive functions like sunlight, assisting in dispelling the darkness and hurdles they encounter.

In studies akin to this one, two essential categories of individual supportive resources such as coping skills and environmental aids like emotional and functional support from families are considered vital for MS patients [15, 16]. Likewise, in a separate study [17], it was discovered that support serves as an antecedent to coping with this disease. In contrast, a study that contradicted this research believes that simply providing spiritual support is enough to address the various challenges experienced during this illness [18]. The variation in the utilization of supportive resources may be linked to patients' past encounters with stressful situations, the diverse backgrounds of individuals with MS, the level of support they receive from their social circles and their access to these resources. In line with the current study, another research [19] highlighted factors that influence the quality of life of MS patients, including individual and interpersonal factors that correspond to the internal and external supports discussed in the current study. The aforementioned study shares methodological similarities with the current study, with differences in participants' age range and age of disease onset, whereas the disease duration was not specified. Given that both studies were conducted in the same country, the congruence in findings likely stems from the comparable social structures among the patients involved in each study. The results of the present study indicate that, despite the profound and widespread impact of the disease on their lives, patients express a sense of well‐being through access to internal and external supportive resources. Another study involving MS patients emphasized the importance of maintaining a positive attitude towards the illness, coupled with utilizing resources and support, to adapt their lifestyles [13]. The varying perspectives among MS patients may be attributed to cultural disparities among participants, with individuals in the aforementioned study likely representing a diverse range of countries, potentially European, with distinct cultural and social frameworks compared to Eastern societies.

The presence of a rainbow is emblematic of achieving vibrant results, representing optimism. Rainbows materialize as a consequence of confronting obstacles and traversing unfamiliar and intimidating paths, such as illness, under the guidance of support. MS can catalyse the advancement of skills, fortify personal strengths, nurture resilience, instil an appreciation for simple moments in life and prompt a shift in perspective towards life, which are indicated as coping features in a study [20]. This study delves into the aftermath of MS, encompassing the restoration of capabilities, personal evolution, finding purpose through resilience and cultivating hope for a brighter tomorrow. These facets inject vibrancy and allure into the lives of patients, furnishing them with enhanced hope and drive to grapple with the disease. As in another study [21], patients tend to have a more positive outlook on life following negative disease‐related experiences.

Resilience emerges from a delicate equilibrium between received support and individual susceptibilities. A recent study has underscored that resilience encompasses coping mechanisms, adaptability, emotional bolstering and overall well‐being [16]. The findings of the current study validate the presence of these facets in prior research. Participants in this study strove to recover their capacities by seeking support, adapting their routines and undergoing psychological and emotional metamorphoses. Through fortifying their existential resilience and employing coping strategies, some individuals experienced personal growth and uncovered significance in their lives by tapping into transcendental wellsprings and delineating personal boundaries. In line with this research, a qualitative study utilizing directional content analysis identified resilience as a central theme through which individuals with MS strive to navigate their condition [22]. It's noteworthy that in the theory being considered, resilience was not predetermined from preset categories. These outcomes may be attributed to similarities in the social and cultural milieus of the patients in both studies. In another study, resilience has been recognized as a key influencer in the quality of life of MS patients [23]. The findings of the current study also underscore the beneficial impacts of resilience on the lives of patients. As resilience burgeoned, hope for the future swelled among patients. These findings may illuminate shared traits among MS patients irrespective of their cultural backgrounds. Despite the prevalent focus in numerous studies on the adversities of living with MS [24, 25, 26, 27], some studies, including the present one, also spotlight positive outcomes. These can encompass enhancements in quality of life, fortified personality traits and the empowerment of individuals to seize opportunities despite encountering myriad challenges [2, 28]. This underscores the notion that even within intricate and unpredictable conditions like MS, positive repercussions are achievable. Strengthened external support systems and coping skills, customized to individuals' unique circumstances, can engender elevated levels of hope and resilience among patients.

## 5 Strengths and Limitations

This study enhances the generalizability of its findings by being conducted in two distinct cities with participants from various backgrounds. It takes a comprehensive approach, exploring all facets of patient experiences, unlike studies that focus on specific aspects of life with MS, disease stages or particular outcomes. One potential limitation is that conversing about upsetting memories with certain patients might evoke distress, potentially causing interviews to halt and offering participants the choice to withdraw.

## 6 Conclusion

In exploring the lives of patients with MS, receiving multidimensional and appropriate support regardless of the severity and type of issues that arise can lead to desirable outcomes. As expressed by the patients in this study, their lives began with the fear of thunderstorm‐like problems and ultimately ended with the emergence of a rainbow of vitality through the support received. Research often tends to focus on the negative aspects of the disease and highlight the suffering of MS patients. However, this study takes a holistic view by exploring all positive and negative aspects of life from the patients' perspective, aiming to enhance understanding through natural and tangible elements.

### 6.1 Recommendations for Further Research

The results of this study provide a foundation for future research aimed at enhancing resilience and hope‐promoting resources. This can serve as a framework for the creation of novel interventions and the evaluation of their effectiveness through interventional studies. Additionally, it is recommended to conduct robust observational studies to assess the potential effects of the existent approach on the health, well‐being and quality of life of individuals living with MS.

### 6.2 Implications for Policy and Practice

The results of this study can assist policymakers and stakeholders in allocating financial resources and designing impactful initiatives that take into account cultural, economic and social factors to prevent and manage the disease. These efforts have the potential to positively influence society and healthcare systems by improving hospital administration, establishing robust support networks, advancing educational initiatives and increasing public awareness.

## Author Contributions

**Leily Zare:** conceptualization, investigation, formal analysis, writing–review and editing, writing–original draft. **Nahid Dehghan Nayeri:** conceptualization, methodology, writing–review and editing. **Fatemeh Bahramnezhad:** conceptualization, methodology, writing–review and editing. **Arezoo Rasti:** conceptualization, supervision, writing–review and editing, project administration.

## Ethics Statement

This research is ethically approved by the Ethics Committee of Tehran University of Medical Sciences with the number IR.TUMS.FNM.REC.1401.185.

## Consent

All participants gave written informed consent before participating in the study.

## Conflicts of Interest

The authors declare no conflicts of interest.

## Supporting information

Supporting information.

## Data Availability

The data supporting the findings could be made available by the corresponding author. The data are not publicly available due to privacy or ethical restrictions.
